# Neuro-Cardio Mechanisms in Huntington’s Disease and Other Neurodegenerative Disorders

**DOI:** 10.3389/fphys.2018.00559

**Published:** 2018-05-23

**Authors:** Bethan J. Critchley, Mark Isalan, Michal Mielcarek

**Affiliations:** ^1^Department of Life Sciences, Imperial College London, London, United Kingdom; ^2^Imperial College Centre for Synthetic Biology, Imperial College London, London, United Kingdom; ^3^Department of Epidemiology of Rare Diseases and Neuroepidemiology, University of Medical Sciences, Poznań, Poland

**Keywords:** neuro-cardio disorders, Huntington’s disease, cardiomyopathy, CNS, heart failure

## Abstract

Although Huntington’s disease is generally considered to be a neurological disorder, there is mounting evidence that heart malfunction plays an important role in disease progression. This is perhaps not unexpected since both cardiovascular and nervous systems are strongly connected – both developmentally and subsequently in health and disease. This connection occurs through a system of central and peripheral neurons that control cardiovascular performance, while in return the cardiovascular system works as a sensor for the nervous system to react to physiological events. Hence, given their permanent interconnectivity, any pathological events occurring in one system might affect the second. In addition, some pathological signals from Huntington’s disease might occur simultaneously in both the cardiovascular and nervous systems, since mutant huntingtin protein is expressed in both. Here we aim to review the source of HD-related cardiomyopathy in the light of recently published studies, and to identify similarities between HD-related cardiomyopathy and other neuro-cardio disorders.

## Introduction

Huntington’s disease (HD), reviewed in Zielonka et al. (2015), is the most common hereditary neurodegenerative disorder. It is invariably fatal and affects approximately 700,000 people worldwide (1 in 10,000). This is likely to be an underestimate of prevalence since a recent screen of healthy individuals reported that 1 in 400 might be at risk of developing HD (Kay et al., 2016). HD is caused by the expansion of a polyglutamine stretch within the huntingtin protein (HTT), which results in the formation of cytotoxic cell products. Poly-CAG DNA repeats in exon1 of the huntingtin (*HTT)* gene code for polyglutamines, and these repeats increase in number in diseased individuals (Walker, 2007). Healthy subjects have fewer than 35 CAG repeats, whereas HD patients range from 35 to ∼200, and repeat number correlates with the age of disease onset (Walker, 2007). In rare cases (∼1 in 100), the HTT gene is apparently not expanded, yet individuals present with clinical manifestations of HD. These cases are classified as HD *phenocopies* – for a summary see Nguyen et al. (2017).

The most widely known feature of HD is neurodegeneration, and this is particularly prevalent in the striatal nuclei, basal ganglia, and cerebral cortex, resulting in neurological symptoms that involve motor, cognitive, and psychiatric disturbances (Zielonka et al., 2015). As a result, patients develop a range of clinical symptoms including personality changes, motor impairment, dementia, and weight loss (Zielonka et al., 2015). In the juvenile form of HD, defined by CAG repeat length of 60 or above and HD diagnosis at 21 years of age or younger, additional symptoms are present, including bradykinesia, muscular hyperkinesis with stiffness, sleep disturbances, tics, moderate to severe leg pain, itching, and psychosis (Moser et al., 2017).

Although brain pathology is the best studied hallmark of HD pathology, HTT is expressed in many other tissues and organs in mammals (Strong et al., 1993). However, to date little is known about the subcellular localization of HTT in CNS and peripheral tissues. Interestingly, HTT exhibits nuclear localization in cultured neuronal cells and cytoplasmic localization in non-neuronal HEK293 cultures, which may be important for its function in different cell types (Tunah, 2012). The protein is an important component in multiple vital cellular processes such as transcription, protein trafficking, and vesicle transport (Li and Li, 2004), hence HD has been identified as a multi-system disorder (Mielcarek, 2015).

The CAG expansion within the HTT gene leads to structural changes within the mutated protein that makes mutant HTT prone to aggregation (Davies et al., 1999). Such aggregation is widespread in the brain and other peripheral tissues (Moffitt et al., 2009) but not in HD hearts (Mielcarek et al., 2014b). Regardless of whether they lead to protein aggregates, HD mutations potentially cause both loss and gain of functions in multifunctional HTT. These may manifest as an altered set of protein interactions; for example, the mutant form of HTT binds to specific histone acetylases (HATs) (Abel and Zukin, 2008) or histone deacetylases, e.g., HDAC4 (Mielcarek et al., 2013). These are global gene regulators which may have a profound effect on gene expression when perturbed, and may be vital for understanding peripheral HD pathologies.

One of the most studied peripheral pathologies in HD is skeletal muscle atrophy (reviewed in Zielonka et al., 2014a; Mielcarek and Isalan, 2015; Mielcarek et al., 2015a, 2017). In fact, a recent study showed that rescuing skeletal muscle degeneration *alone* had a profound therapeutic effect in HD model mice. This was achieved using myostatin inhibition, resulting in reduced loss of muscle mass and grip strength impairment during the expected life span of an R6/2 mouse. More importantly, it delayed onset of end-stage HD by approximately 20% of the R6/2 mouse life span (Mielcarek et al., 2014c). Since the pharmacological intervention took place specifically in skeletal muscle, i.e., peripherally to the CNS, these findings show that in principle the CNS-skeletal muscle axis can be successfully targeted on the peripheral end. This underlines the importance of peripheral pathology as a potential stand-alone therapeutic target in HD.

In this review, we aim to concentrate on the source of HD-related cardiomyopathy. We summarize pathological cardiovascular events in HD mouse models, both in the pre-symptomatic phase, when no behavioral abnormalities are present, and the symptomatic phase, where behavioral and molecular events are fully present. This is followed by an exploration of the cardiovascular abnormalities present in the symptomatic stage of disease in human patients. Finally, we examine similarities between HD-related cardiomyopathy and the cardiovascular component of other neurodegenerative diseases.

## Heart Pathological Events in Pre-Symptomatic HD Mouse Models

The biological function of HTT remains unknown not only in the heart, but also in other tissues including the CNS. However, proof-of-concept studies underpinning mutant HTT toxicity in the heart have been performed in various animal models. An artificial transgenic polyQ-expressing mouse model has been reported to develop heart failure, which led to premature death at eight months of age (Pattison et al., 2008). These mice expressed either a 19-glutamine control polyQ peptide (PQ19), or an 83-glutamine mutant polyQ peptide (PQ83), under regulatory control of the α-myosin heavy chain promoter (MyHC) which drives cardiomyocyte-specific expression. The results appear to indicate an intrinsic toxic function of mutant HTT in HD hearts (Pattison et al., 2008), despite no wide-spread CNS pathology.

A similar study was performed in a *Drosophila* model where mutant exon-1 HTT was expressed under the control of the Hand1 (heart) promoter. This model developed an increased incidence of arrhythmias and extreme cardiac hypertrophy, accompanied by a significant decrease in contractility (Melkani et al., 2013).

Both these models clearly indicate an intrinsic toxic function of mutant HTT in HD hearts that is independent of CNS pathology. However, these experimental conditions are somewhat different from the natural pathology (where no high molecular weight aggregates are observed; Mielcarek et al., 2014b), since both models highly over-expressed either polyQ(83) peptide or mutant exon-1 HTT in cardiac tissue. On the other hand, a number of human heart failure samples of various etiologies have shown accumulation of intracellular pre-amyloid oligomers and higher-order assemblies (Sanbe et al., 2005). In a model of desmin-related cardiomyopathy, produced by expressing the mutant form of the cardiomyocyte-specific transgene αB-crystallin (CryAB^R120G^), aggregates were observed within cardiomyocytes which led to altered cardiomyocyte function, perturbations in mitochondrial-sarcomere architecture, and deficits in mitochondrial function, which collectively caused apoptosis and heart failure (Maloyan et al., 2005).

**FIGURE 1:**
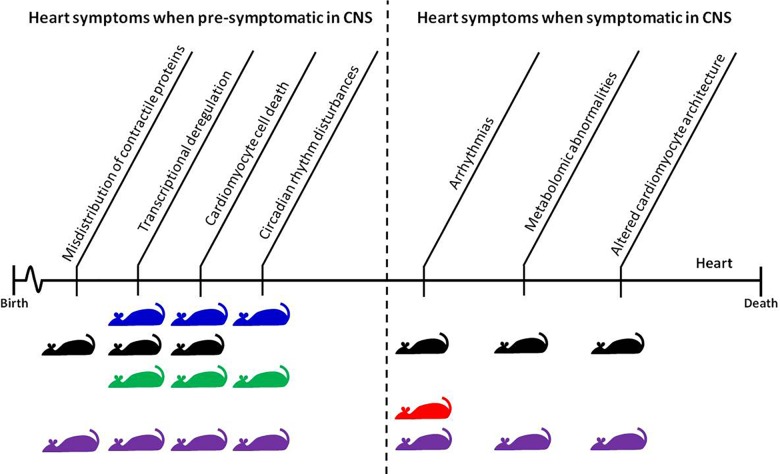
Time-dependent appearance of molecular and physiological features of HD-related cardiomyopathy and their corresponding mouse models. The CNS pre-symptomatic stage displays no obvious molecular and physiological abnormalities in neuronal pathology and no detectable behavioral abnormalities. However, pathological events already begin to present and elevate in the heart during this phase. The symptomatic and end-stage of disease corresponds with full penetrance of HD-related pathology in the CNS, and significant changes in behavioral readouts in pre-clinical settings. Mouse models: blue, BACHD; black, *Hdh*Q150; green, Q175; red, R6/1; purple, R6/2.

In HD mouse models, several groups published data describing heart pathological events in symptomatic animals and at the end-stage of disease (**Figure 1**). However, there is growing evidence that some pathological events in HD hearts occur prior to CNS degeneration. For example, it has been shown that Connexin-43 in gap junctions dislocates from the end plate towards the lateral membrane in cardiomyocytes as early as 4 weeks of age in R6/2 mice, and 8 months of age in *Hdh*Q150 mice, while its protein levels remained unchanged (Mielcarek et al., 2014b). This might be a first link to conduction disturbances and arrhythmogenesis in many heart diseases (Delmar and Makita, 2012). It will be vital for future therapeutic avenues to validate whether mutant HTT also alters expression/distribution of other heart contractile proteins (Yin et al., 2015). The connexin-43 relocation was accompanied by a significant deregulation of hypertrophic markers and *Bdnf* transcripts, a significant degree of fibrosis, and an increased level of apoptotic nuclei in pre-symptomatic mouse models (Mielcarek et al., 2014b). In the BACHD mouse model, the first structural and functional differences (i.e., end systolic dimension) have been detected in pre-symptomatic animals as early as 3 months of age, and continued throughout their life span. At this early stage, HD mice displayed profound transcriptional changes related to key biological process including apoptosis, gene expression, proliferation, and proteolysis/ubiquitination (Schroeder et al., 2016). In addition, the young mice showed evidence of an upregulated pro-inflammatory immune response, particularly with respect to IL-6 levels, which could contribute to the observed cardiopathological changes (Schroeder et al., 2016).

Recently, another pathological component of the cardiovascular system has been identified pre-symptomatically in the R6/2 mouse model. These mice showed a significant enhancement of endothelial-dependent dilation and impairment of α1 adrenergic vasoconstrictor responses. Consequently, the mice failed to recruit NO-dependent pathways and displayed increased endothelial-mediated relaxation. In addition, symptomatic R6/2 mice had significantly altered endothelial function in peripheral resistance arteries (Kane et al., 2016). Moreover, μMRA (three-dimensional microscopic magnetic resonance angiography) revealed an increase in vessel volume fraction and cerebral blood volume in the brains of pre-symptomatic R6/2 mice. Collagen IV immunostaining revealed an enrichment in vessel density, but no increase in vessel size, within the microvasculature of the mouse HD brain, which worsened with disease progression. Most importantly, vessel densities in the cortex, caudate/putamen, and substantia nigra were higher in early symptomatic HD patients compared to non-HD human subjects (Lin et al., 2013). Taken together, these data suggest a link between HD and changes in vasculature.

In addition, symptomatic Q175 knock-in mice demonstrate disturbances to circadian and diurnal rhythms, including blunted daily cycling in heart rate and heart rate variability as well as autonomic dysfunction (demonstrated by lack of core body temperature regulation and poor performance in the baroreceptor challenge) (Cutler et al., 2017). Similar circadian disturbances have been seen in BACHD and R6/2 mice (Kudo et al., 2011). In conclusion, there is mounting evidence that circadian rhythms are impaired both in HD mouse models and in HD patients; this may have profound consequences for heart malfunction with the source being the CNS axis end.

## Heart Pathological Events in Symptomatic HD Mouse Models

Recently, several studies have examined heart malfunction in the symptomatic and late-end stage of HD using different HD mouse models which are characterized by time-dependent disease progression (see **Figure 1** for an overview). Early pathological events in the heart such as connexin-43 dislocation, transcriptional deregulation, and apoptotic cell death were elevated throughout the life span of R6/2 and *Hdh*Q150 mouse models (Mielcarek et al., 2014b). This was accompanied by an increased level of fibrotic deposits in both R6/2 and *Hdh*Q150 (Mielcarek et al., 2014b). In Q175 knock-in mice, pockets of fibrosis were observed in the interventricular septum, and the size of cardiomyocytes was reduced, both of which exhibited age-dependent progression (Cutler et al., 2017). Similarly, symptomatic BACHD mice develop cardiac fibrosis and ultimately apoptosis (Schroeder et al., 2016). In fact, cardiac Fas-dependent and mitochondria-dependent apoptotic pathways have been identified to be activated in the hearts of symptomatic R6/2 mice. This was accompanied by increased levels of crucial components of both Fas-dependent apoptosis (TNF-alpha, TNFR1, Fas ligand, Fas death receptors, FADD, activated caspase-8, and activated caspase-3) and mitochondria-dependent apoptosis (Bax, Bax-to- Bcl-2 ratio, cytosolic cytochrome c, activated caspase-9, and activated caspase-3) (Wu et al., 2015). Furthermore, during disease progression, the number of transcripts with altered expression increased significantly in R6/2 and *Hdh*Q150 mouse models (Mielcarek et al., 2014b) as well in BACHD mice (Schroeder et al., 2016). Therefore cardiac molecular changes are observed across many HD mouse models.

In symptomatic HD mouse models, pronounced functional changes were visualized by cardiac MRI, revealing a number of abnormalities. These may contribute to dilated cardiomyopathy (DCM) in the R6/2 mouse model (Wood et al., 2012) and *Hdh*Q150 mice (Mielcarek et al., 2014b). At the physiological level, HD hearts also displayed a contractile dysfunction based on ECG measurements in two HD mouse models (R6/2 and *Hdh*Q150) (Mielcarek et al., 2014b). This was supported by molecular pathological events including altered architecture of ganglionic plexuses (observed using tyrosine hydroxylase staining) and lower levels of *Bdnf* transcripts in HD murine hearts (Mielcarek et al., 2014b). The R6/1 model, which had unstable RR intervals that were reversed following atropine treatment, shows similar molecular pathological events, suggesting parasympathetic nervous activation. The mice developed brady- and tachyarrhythmias, including paroxysmal atrial fibrillation and cardiac sudden death (Kiriazis et al., 2012).

Since myocardial contraction depends strongly on mitochondrial energy supply, it is likely that HD murine hearts could display alterations in cardiac energy equilibrium. Consequently, an investigation of both R6/2 and *Hdh*Q150 mouse models showed that they have reduced glucose usage and a significant deregulation of genes involved in *de novo* purine biosynthesis, conversion of adenine nucleotides, and adenosine metabolism (despite AMP-activated protein kinase hyperphosphorylation) (Toczek et al., 2016b). At the molecular level, increased levels of nucleotide catabolites such as inosine, hypoxanthine, xanthine, and uric acid and decreased levels of adenosine have been observed in murine and human HD serum. These metabolites represent the first identified biomarkers related to striated muscle dysfunction in HD, in both pre-clinical and clinical settings (Toczek et al., 2016b). High-performance liquid chromatography assays demonstrate that enzymatic activity is also affected; AMPD and e5′NT exhibit decreased activity whereas ADA activity levels are increased, suggesting that mutant HTT could be disrupting cardiac nucleotide metabolism on a transcriptional level (Toczek et al., 2016a). Importantly, mutant HTT interacts with global gene regulators, such as HDACs, and this is potentially important for the gene expression changes observed in HD, as well as other types of cardiomyopathies (Mielcarek et al., 2013, 2015b; Piotrowska et al., 2017).

Rodent heart disease models also exhibit alterations in cardiac energy equilibrium, with rat aortic constriction models showing reduced glucose metabolism similar to R6/2 and *HdH*Q150 HD models (Doenst et al., 2013). Furthermore, models of myocardial infarction, hypertension, renal failure, and aortic constriction provide evidence for mitochondrial dysfunction or deregulated mitochondrial gene expression (Doenst et al., 2013). Overall, these similarities between rodent models of HD and heart failure do suggest that HD-related pathology could contribute to heart failure due to the disruption of the cardiac energy balance.

## HD Related Cardiomyopathy in the Clinic

Since Huntington’s disease has been described in the literature as an exclusively neurological disorder, there are a very limited number of studies evaluating heart function in HD patients. This is despite previously published epidemiological data that clearly identified heart failure as the second most common cause of death in HD (reviewed in Zielonka et al., 2014b). Only very recently, a clinical study by Stephen et al. (2015) on a large cohort of early symptomatic patients revealed significant contractile heart dysfunction. The study was performed using standard 12-lead electrocardiograms (ECGs) and found that abnormal ECGs were typical for 25.3% of early symptomatic patients. Abnormalities were manifested in several ways, including bradycardia and prolonged QTc interval and/or intra-ventricular conduction, likely leading to arrhythmia and aggravated cardiac failure. An additional study on 41 HD subjects found significantly greater arterial stiffness (as a result of diminished vascular smooth muscle contractility due to autonomic dysfunction) in pre-symptomatic and early-stage HD as well as increased intima media thickness in mid and late stages (Kobal et al., 2017). These findings support those from multiple studies of different HD mouse models, which also revealed contractile heart dysfunction at various stages of disease progression (Kiriazis et al., 2012; Mielcarek et al., 2014b; Schroeder et al., 2016).

In addition, several clinical reports performed on small cohorts of early or symptomatic patients revealed that HD patients have enhanced cardiovagal activity (Andrich et al., 2002), reduced heart rate (Melik et al., 2012), and 10.6% lower diastolic pressure (Kobal et al., 2010). Unfortunately, to date there is no molecular data available that may underpin the mechanism of heart failure in human HD. The only study published so far identified a set of nucleotide catabolites in human HD sera which are associated with failing HD hearts (Toczek et al., 2016b).

## Shared Pathological Events in HD-Related Cardiomyopathy and Other Cardio-Neuro Diseases

**FIGURE 2:**
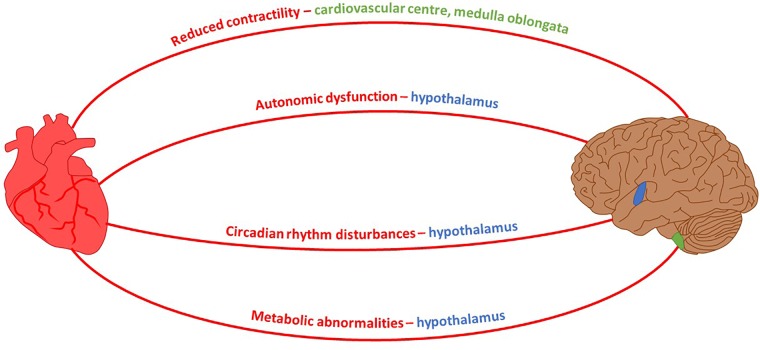
Neuro-Cardio connections between HD-related cardiomyopathy and brain regions affected by HD. Mouse model HD symptoms and their associated brain control region which exhibits degeneration during the course of HD pathology. Shaded regions of the brain: blue, hypothalamus; green, medulla oblongata (Petersen and Gabery, 2012; Rub et al., 2014).

It is well known that the canonical approach to studying any neurodegenerative disease focuses exclusively on neuronal defects caused by inherited or non-inherited factors. This is against a school of thought suggesting a permanent interconnectivity between the cardiovascular and nervous systems. Since both systems are directly linked, any pathological insult causing malfunction in one of them may have an indirect impact on the other. Multiple neuro-cardio diseases demonstrate their interconnectivity – Huntington’s disease serves as a good example because several of the cardiac dysfunctions which present over the course of disease progression are controlled by brain regions affected during HD pathology (**Figure 2**). As such, an approach towards finding novel cardiac therapeutic avenues might at least delay HD symptoms.

There are numerous hereditary and non-hereditary neurodegenerative diseases, in which cardiovascular malfunction has been identified as a leading element in disease progression, often manifesting as arrhythmias, cardiomyopathy, or autonomic dysfunction. Cardiomyopathy is the most common cause of death and affects 66% of patients with FA (Friedreich’s ataxia), an autosomal recessive neuro and cardio-degenerative disorder that is caused by deficient expression of the nuclear-encoded mitochondrial protein frataxin (Lane et al., 2013). Patients often present with left ventricular hypertrophy, which can develop into more fatal dilated cardiomyopathy (Lane et al., 2013). Moreover, FA cardiomyocytes are significantly larger than control cells and are surrounded by fibrotic endomysium (Koeppen et al., 2015). There are numerous mouse models of FA that develop heart cardiomyopathy reflecting clinical endpoints (Payne et al., 2011). Similarly, large numbers of fibrotic deposits and cardiomyocyte disarray has been identified in pre-clinical models of HD (Mielcarek et al., 2014b; Schroeder et al., 2016).

Neurodegenerative disorders like Parkinson’s disease (PD) (Dauer and Przedborski, 2003; Amino et al., 2005) are often accompanied by fatigue, dysrhythmias, and dysregulation in the electrical activity, i.e., prolongation in the corrected QTc interval or reduced heart rate variability (reviewed in Joers and Emborg, 2014). Interestingly, although originally described as a movement disorder linked to the loss of dopaminergic neurons, PD has recently been classified as a multisystem disorder affecting both central and peripheral nervous systems (Dauer and Przedborski, 2003; Amino et al., 2005). Arrhythmias have also been observed in bulbar spinal muscular atrophy (BSMA), which could be caused by myocardial accumulation of mutant androgen receptor protein, analogous to Lewy body formation in PD neurons (Araki et al., 2014). There is also evidence to suggest that Lewy bodies occur in epicardial nerve fascicles prior to CNS degeneration (Navarro-Otano et al., 2013), and similar findings have been reported in HD pre-clinical and clinical settings (Mielcarek et al., 2014b; Stephen et al., 2015).

Familial Alzheimer’s disease (AD) is predominantly caused by presenilin 1 (*PSEN1*) or presenilin 2 (*PSEN2*) mutations (Bird, 2005). The presenilins are also expressed in the heart and have been found to be critical to cardiac development (Donoviel et al., 1999; Nakajima et al., 2004). *PSEN1* mutation has been associated with complete penetrance and progressive disease that necessitates cardiac transplantation or results in death (Li et al., 2006). By contrast, *PSEN2* mutation results in partial penetrance, milder disease, and a more favorable prognosis. Nonetheless, it is striking that both mutations are associated with a higher risk of dilated cardiomyopathy and heart failure in AD patients (Li et al., 2006).

AD also has a cardiovascular defect (similar to HD) associated with microvascular dysfunction and/or neurovascular disintegration. Microvascular deficits diminish CBF and, subsequently, the brain’s supply of oxygen, energy substrates, and nutrients (reviewed in Guo and Lo, 2009; Zlokovic, 2011).

In terms of signaling, the APP/PS1 AD model shows reduced responsiveness to adrenergic agonists, although β_1_-adrenergic receptor expression is apparently unchanged in APP/PS1 hearts (Turdi et al., 2009). This is similar to the lack of responsiveness to β_1_-adrenergic receptor stimulation which has been observed in symptomatic HD R6/2 mice when chronically treated with isoproterenol, a potent beta-adrenoreceptor agonist (Mielcarek et al., 2014a). Moreover, representative heart sections in the primary literature show BACHD symptomatic mouse hearts, treated with isoproterenol, as having extremely high levels of fibrosis, much more than would be expected with this drug treatment (Schroeder et al., 2016). In this case, the heart muscle appears to be dilated as opposed to hypertrophic. Pre-symptomatic BACHD mice respond adaptively to isoproterenol treatment, suggesting that response failure to adrenergic signaling only manifests once behavioral and molecular changes have emerged (Schroeder et al., 2016).

Finally, Timothy syndrome (TS) is a multi-system disorder which affects both the nervous and cardiac systems and is caused by a missense point mutation in the CACNA1C gene (Dixon et al., 2012). Similar to HD, patients suffer from a prolonged QT interval and structural heart defects in addition to neurodevelopmental delay, seizures, and autism-spectrum disorder symptoms (Splawski et al., 2004). Other neuro-cardio diseases may exhibit partially penetrant symptom crossover with HD cardiac pathology. For instance, around 50% of patients with Amyotrophic lateral sclerosis (ALS) may be affected by chronic cardiac sympathetic hyperactivity, which is linked to sudden cardiac death and stress-induced cardiomyopathy – similar to the cardiomyopathy seen in pre-clinical and clinical HD (Tanaka et al., 2013). Additionally, ALS patients have elevated resting blood pressure and, in contrast to the diminished response to β_1_-adrenergic receptor stimulation in HD and AD patients, exhibit increased response to alpha-adrenergic stimulation via administration of phentolamine (Tanaka et al., 2013).

## Conclusion

**FIGURE 3:**
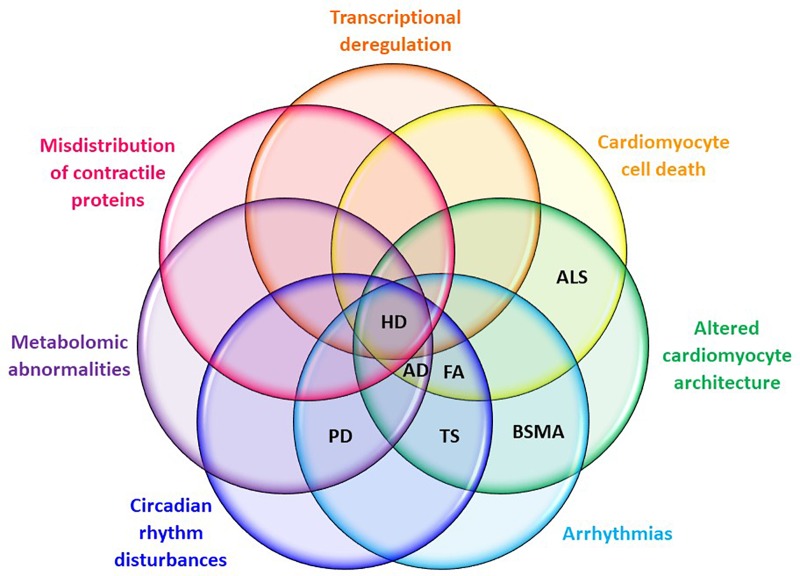
Cardiac symptom overlaps between HD and other neuro-cardio diseases. Symptoms listed are those evident in mouse models of HD at CNS pre-symptomatic or symptomatic stages. AD, Alzheimer’s disease; ALS, amyotrophic lateral sclerosis; BSMA, bulbar spinal muscular atrophy; FA, Friedreich’s ataxia; HD, Huntington’s disease; PD, Parkinson’s disease; TS, Timothy syndrome.

Huntington’s disease shares many pathological features with other neurodegenerative disorders, including peripheral organ malfunction in the heart (see **Figure 3** for a summary of shared cardiopathological symptoms). Recent studies provide new and strong evidence that HD-related cardiomyopathy plays a detrimental role in disease progression. However, urgent studies are now needed to fully understand not only the pathological consequences of HD-related cardiomyopathy, but also to underpin its molecular source using genetic approaches. This new understanding will be beneficial in order to fully ameliorate all pathological features of HD, allowing the development of new therapeutic strategies.

## Author Contributions

All authors listed have made a substantial, direct, and intellectual contribution to the work, and approved it for publication.

## Conflict of Interest Statement

The authors declare that the research was conducted in the absence of any commercial or financial relationships that could be construed as a potential conflict of interest.
